# Hypoplasia of the inferior vena cava with compensatory azygos–hemiazygos dilatation complicated by superior mesenteric vein thrombosis and small bowel ischemia

**DOI:** 10.1016/j.jvscit.2026.102233

**Published:** 2026-03-20

**Authors:** Majd Oweidat, Yaman Abd Alfatah, Noor Khderat, Eman AL-Najjar, Malak Amro, Bushra Ibrahim Atea, Haya Taha, Mohammed Maraqa, Sulaiman Naji Fares Fakhouri, Ahmad Thiab Al-baw

**Affiliations:** a College of Medicine, Hebron University, Hebron, West Bank, Palestine; b Palestine Red Crescent Specialized Hospital - Hebron, Hebron, West Bank, Palestine; c College of Medicine and Health Sciences, Palestine Polytechnic University, Hebron, West Bank, Palestine; d Department of Radiology, Princess Alia Hebron Governmental Hospital, Hebron, West Bank, Palestine; e Department of Surgery, Princess Alia Hebron Governmental Hospital, Hebron, West Bank, Palestine

**Keywords:** Acute mesenteric ischemia, Azygos–hemiazygos dilatation, Inferior vena cava hypoplasia, Mesenteric venous thrombosis

## Abstract

Congenital anomalies of the inferior vena cava (IVC) are rare and often clinically silent but may predispose to venous thrombosis through altered hemodynamics. We report a woman in her late sixties who presented with acute severe abdominal pain and shock physiology. Contrast-enhanced computed tomography showed hypoplasia of the infrahepatic IVC with marked azygos–hemiazygos continuation, complicated by extensive superior mesenteric vein thrombosis and ischemic ileal bowel. Despite aggressive resuscitation and emergent surgical planning, the patient rapidly deteriorated and died. This case highlights IVC hypoplasia as a potential predisposing factor for catastrophic mesenteric venous thrombosis.

The inferior vena cava (IVC) is the principal retroperitoneal conduit returning deoxygenated blood from the abdomen and lower extremities to the right atrium. It forms from the confluence of the common iliac veins (typically at L5) and relies on respiratory pressure gradients to facilitate forward flow.^1^ Importantly, the ascending lumbar veins connect the lumbar venous network to the azygos system, providing a potential collateral pathway between the IVC and superior vena cava. This collateral capacity becomes clinically relevant when the IVC is congenitally malformed or obstructed.^1^

The azygos vein, formed by the junction of the right subcostal and ascending lumbar veins, ascends within the posterior mediastinum to drain into the superior vena cava and functions as a key collateral channel between the two caval systems.^2^ In congenital interruption or failed development of segments of the IVC, venous return from the lower body may be diverted through a dilated azygos–hemiazygos system (“azygos continuation”). Although such developmental variants are typically incidental, they have procedural implications and may predispose to venous stasis and thromboembolic disease.^2^

Mesenteric venous thrombosis (MVT) is an uncommon but high-stakes cause of acute mesenteric ischemia, accounting for approximately 5% to 15% of mesenteric ischemic events; the superior mesenteric vein (SMV) is involved in most cases. Clinical manifestations range from abdominal pain to hemodynamic instability and septic shock, and delays in diagnosis and treatment substantially worsen outcomes.^3^^,^^4^

This report describes a novel association: hypoplasia of the infra-hepatic IVC with compensatory azygos–hemiazygos dilatation complicated by acute SMV thrombosis (SMVT) and small bowel ischemia.

## Case report

A woman in her late sixties presented to the emergency department with a 1-day history of progressively worsening lower abdominal pain that was constant, severe, and not related to meals. She reported repeated vomiting and several episodes of diarrhea, and denied symptoms suggestive of an underlying malignancy, constipation, fever, recent trauma, prior abdominal operations, and use of hormonal therapy. Her medical history was notable for coronary artery disease treated with coronary artery bypass grafting more than a decade earlier. She did not smoke, reported no substance use, and had no personal or family history of diabetes mellitus, hypertension, or known thrombophilia. Her regular medications were acetylsalicylic acid 10 mg once daily and bisoprolol 2.5 mg once daily.

On arrival, she appeared distressed and clinically dehydrated, with a temperature of 38.0°C, blood pressure 90/55 mmHg, heart rate 100 beats per minute, and respiratory rate 25 breaths per minute. Abdominal examination showed minimal tenderness, maximal in the lower abdomen, without guarding, rigidity, or rebound tenderness; bowel sounds were reduced, and there was no abdominal distension. Cardiopulmonary and neurological examinations were otherwise unremarkable, and a comprehensive review of systems did not identify chest pain, dyspnea, urinary symptoms, focal neurological deficits, or skin changes beyond those consistent with dehydration. An electrocardiogram was unremarkable.

Given the severity of pain with relatively limited abdominal signs and the presence of shock physiology, acute mesenteric ischemia was suspected; resuscitation was initiated in parallel with urgent imaging and did not delay it. She received supplemental oxygen, two large-bore peripheral intravenous (IV) cannulas were placed, and aggressive IV fluid resuscitation was started while blood tests were obtained.

**Fig 1:**
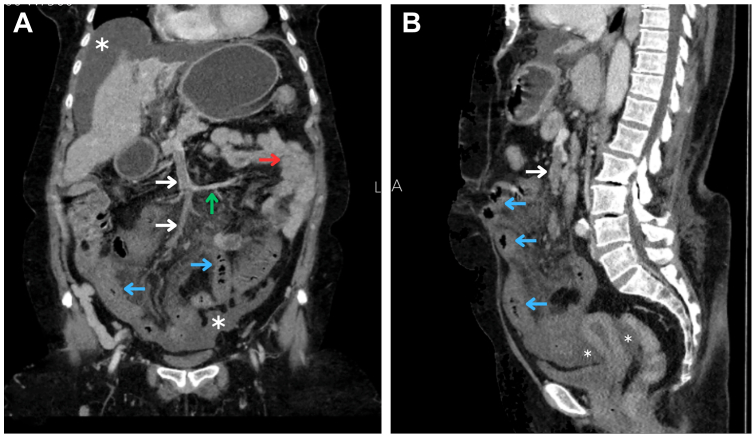
Contrast-enhanced abdominal computed tomography angiography (CTA) images in coronal **(A)** and sagittal **(B)** planes demonstrate circumferential wall thickening of the ileal loops (*blue arrows*) compared with normally enhancing jejunal loops in the left upper quadrant (*red arrowhead*). A filling defect is noted in the superior mesenteric vein (SMV) and its tributaries (*white arrows*), whereas the jejunal vein remains patent (*green arrow*). Associated free fluid is present in the abdomen and pelvis (*white asterisk*).

**Fig 2:**
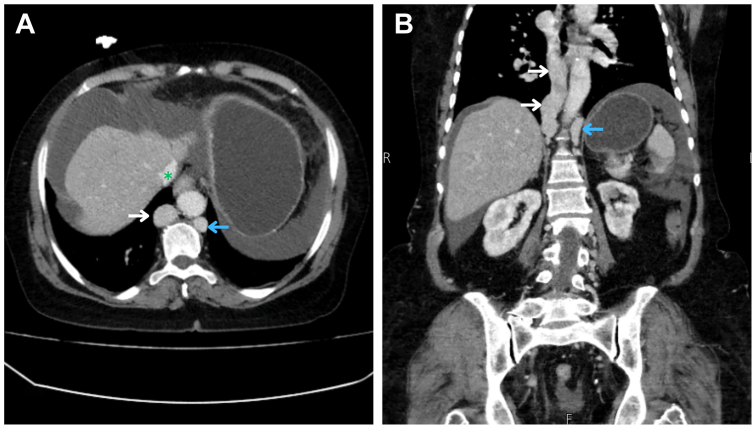
Contrast-enhanced axial **(A)** and coronal **(B)** computed tomography angiography (CTA) images show dilated azygous (*white arrow*) and hemiazygos (*blue arrow*) veins, with a normal-caliber supra-hepatic inferior vena cava (IVC; *green asterisk*, axial image **[A]**).

**Fig 3:**
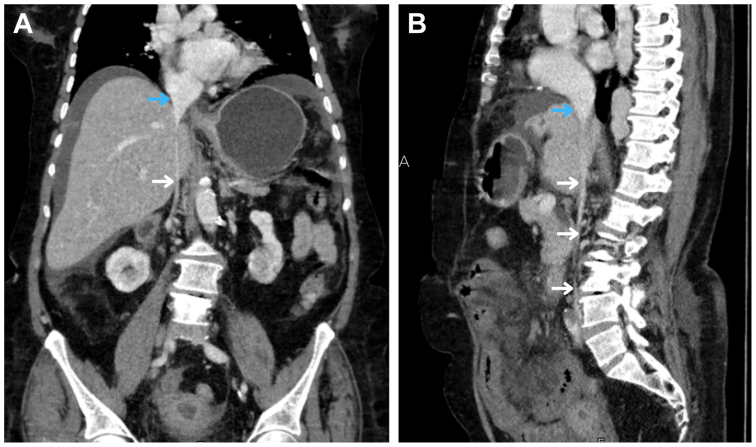
Contrast-enhanced abdominal computed tomography angiography (CTA) images in coronal **(A)** and sagittal **(B)** planes demonstrate a normal-caliber supra-hepatic inferior vena cava (IVC; *blue arrows*) followed by small-caliber but patent infra-hepatic IVC (*white arrows*). On the sagittal plane **(B)**, the entire length of the IVC is visualized extending from its origin at the iliac bifurcation to its entry into the right atrium.

A triphasic contrast-enhanced computed tomography angiography (CTA) of the abdomen and pelvis showed hypo-enhancing circumferential wall thickening involving a long segment of the ileal bowel loops, associated with congested mesentery (Fig 1). A filling defect was noted within the SMV, extending into the ileal and ileocolic branches while sparing the jejunal branch. A moderate amount of free fluid was observed within the abdomen and pelvis. These findings are suggestive of ischemic ileal bowel secondary to SMV thrombosis. Additionally, the azygous and hemiazygos veins were markedly dilated throughout the thoracic and abdominal regions (Fig 2). The infra-hepatic IVC appeared hypoplastic, including its tributaries such as the renal veins and common iliac veins (Fig 3). Anastomotic collateral channels were identified between the azygos–hemiazygos system and the hypoplastic renal and iliac veins. The right superficial circumflex iliac vein was dilated and demonstrated a filling defect near its distal segment. The hepatic venous outflow was patent without radiologic evidence of stenosis or portal hypertension. No radiologic features suggestive of hepatopancreatobiliary malignancy or intraabdominal inflammatory or infectious pathology were identified.

She was admitted to the intensive care unit for continued hemodynamic stabilization. Management included ongoing aggressive IV fluids, nasogastric decompression, broad-spectrum IV antibiotics, and immediate initiation of therapeutic anticoagulation with a weight-based bolus of unfractionated heparin. Initial laboratory results showed leukocytosis of 30.4 × 10^3^/μL and hemoglobin of 18.2 g/dL. Arterial blood gas analysis revealed severe metabolic acidosis with pH 7.15, PaCO_2_ 33 mmHg, HCO_3_^-^ 11.5 mmol/L, PaO_2_ 34 mmHg, and serum lactate 7.2 mmol/L. D-dimer was 10,944 ng/mL, creatinine 1.8 mg/dL, and blood urea nitrogen 28 mg/dL. Otherwise, laboratory tests were within the normal limits, including thrombophilia screening and antiphospholipid antibody testing. However, given the presence of hemodynamic instability, severe metabolic acidosis, elevated serum lactate, and concern for established bowel infarction, immediate exploratory laparotomy was prioritized by the surgical team over other management strategies. There was no delay in operative decision-making; immediately following CTA confirmation of extensive SMVT, the patient was prepared for urgent transfer to the operating room.

Despite the above-mentioned measures, she suffered cardiac arrest immediately prior to transfer to the operating room and died despite the resuscitative efforts.

Informed consent for publication of case details and images was obtained from the patient’s family.

## Discussion

Congenital IVC anomalies arise from complex embryologic remodeling and may manifest as hypoplasia, interruption, duplication, or agenesis. Although most are detected incidentally, the azygos–hemiazygos system can become the dominant venous return route when the IVC is absent or interrupted, producing marked paravertebral venous enlargement that may mimic mediastinal pathology on chest radiography and complicate endovascular or cardiothoracic procedures.^1^^,^^5^ Recognition is therefore clinically consequential, including avoiding catastrophic outcomes such as inadvertent ligation of the azygos vein when it represents the only major drainage pathway from below the diaphragm.

Reviewing some published cases, well-developed azygos–hemiazygos compensation often permits an asymptomatic course, yet altered venous anatomy may increase caudal venous pressures and promote stasis, predisposing to thrombosis. Mandato et al emphasized that azygos continuation can be associated with thrombotic events in patients with otherwise unexplained deep venous thrombosis.^6^ Similarly, Hamoud et al reported IVC hypoplasia with azygos continuation presenting as recurrent leg deep vein thrombosis.^7^ Lluis Pons et al reported IVC agenesis associated with simultaneous mesenteric-portal axis thrombosis and multiple venous thromboses.^8^ More recently, Montatore et al described subrenal IVC hypoplasia with hypertrophic azygos–hemiazygos veins and prominent anterior abdominal wall collaterals in an asymptomatic patient.^9^ Additionally, thrombotic events may occur to an asymptomatic patient when additional acquired risk factors supervene. In this case, dehydration and age-related endothelial vulnerability may have acted as precipitating factors in the setting of pre-existing altered venous hemodynamics.

In acute MVT, symptoms may be nonspecific yet severe, including pain out of proportion to examination and progression to hemodynamic instability. Contrast-enhanced CTA is the preferred diagnostic modality and can show venous filling defects as well as ischemic features such as bowel wall thickening, mesenteric edema, and free fluid.^4^^,^^10^ In the present case, CTA identified extensive venous thrombosis involving the SMV branches with imaging features of small-bowel ischemia in the setting of azygos–hemiazygos dilatation and a hypoplastic infra-hepatic IVC.

Current guidance for acute/subacute MVT prioritizes rapid resuscitation and early anticoagulation (often unfractionated heparin when instability or potential intervention is anticipated), with antibiotics when concern exists for bacterial translocation or thrombophlebitis, and urgent surgery when bowel infarction or sepsis is suspected.^11^^,^^12^ The fulminant deterioration in this case aligns with the recognized high mortality of mesenteric ischemia when diagnosis is delayed or when advanced ischemia is present at presentation.

Taken together, this anatomic variant likely created a hemodynamic environment compatible with venous stasis within the splanchnic circulation and may have acted as a predisposing factor for the SMVT observed in this patient. Although causality cannot be proven from a single case, this association warrants consideration when evaluating unexplained MVT, as it may influence the management strategies.

## Conclusions

Congenital IVC hypoplasia with azygos–hemiazygos continuation may predispose to catastrophic MVT.

## Funding

None.

## Disclosures

None.
